# XenoCell: classification of cellular barcodes in single cell experiments from xenograft samples

**DOI:** 10.1186/s12920-021-00872-8

**Published:** 2021-01-29

**Authors:** Stefano Cheloni, Roman Hillje, Lucilla Luzi, Pier Giuseppe Pelicci, Elena Gatti

**Affiliations:** 1 Department of Experimental Oncology, IEO, European Institute of Oncology IRCCS, Via Adamello 16, 20139 Milan, Italy; 2 Department of Oncology and Hemato-Oncology, Università Degli Studi Di Milano, Milan, Italy

**Keywords:** Single-cell, Xenografts, Transcriptomics, Genomics, Next-generation sequencing

## Abstract

**Background:**

Single-cell sequencing technologies provide unprecedented opportunities to deconvolve the genomic, transcriptomic or epigenomic heterogeneity of complex biological systems. Its application in samples from xenografts of patient-derived biopsies (PDX), however, is limited by the presence of cells originating from both the host and the graft in the analysed samples; in fact, in the bioinformatics workflows it is still a challenge discriminating between host and graft sequence reads obtained in a single-cell experiment.

**Results:**

We have developed XenoCell, the first stand-alone pre-processing tool that performs fast and reliable classification of host and graft cellular barcodes from single-cell sequencing experiments. We show its application on a mixed species 50:50 cell line experiment from 10× Genomics platform, and on a publicly available PDX dataset obtained by Drop-Seq.

**Conclusions:**

XenoCell accurately dissects sequence reads from any host and graft combination of species as well as from a broad range of single-cell experiments and platforms. It is open source and available at https://gitlab.com/XenoCell/XenoCell.

## Background

Patient-derived xenografts (PDX) are being increasingly recognized as relevant preclinical models in many areas of biomedical research, including oncology and immunology. In recent years, the development and rapid diffusion of ultra-high-throughput droplet-based single-cell (sc) sequencing technologies has allowed resolution of genomic, transcriptomic and epigenomic profiles at the level of individual cells [1]. This approach proved to be invaluable for the analyses of complex and/or heterogenous biological systems, and will be increasingly used to analyse human xenograft samples [2].

One potential limit of single-cell sequencing experiments of xenograft samples is the presence of host (e.g. mouse) cells along with graft (e.g. human) cells. Moreover, for reasons that are inherent to the droplet technology, a cell originating from the host may accidentally be encapsulated in the same droplet of a cell from the graft, forming a mixed-species multiplet. While several solutions have been proposed for the identification of multiplets [3, 4], few approaches are available to reduce host-cell contamination. Contamination may be reduced using upstream physical or biochemical strategies such as flow cytometry-based cell sorting or laser microdissection. Downstream in silico techniques have been developed to separate human-mouse chimeric data by classifying individual reads, but are limited to NGS experiments on bulk cell populations [5, 6]. To date, there are no stand-alone tools available to pre-process chimeric data from ultra-high-throughput droplet-based single-cell sequencing experiments of xenograft samples. Here, we propose XenoCell to overcome this challenge, by extending the functionality of Xenome [5] for the classification of individual droplets and separation of cells from different organisms.

## Implementation

### The XenoCell workflow

XenoCell is implemented in Python and made available as a Docker image, which contains all third-party software dependencies as a pre-configured system in order to facilitate portability across platforms and easier integration into existing workflows, such as those built with the Snakemake [7] or Nextflow [8] frameworks.

**Fig. 1:**
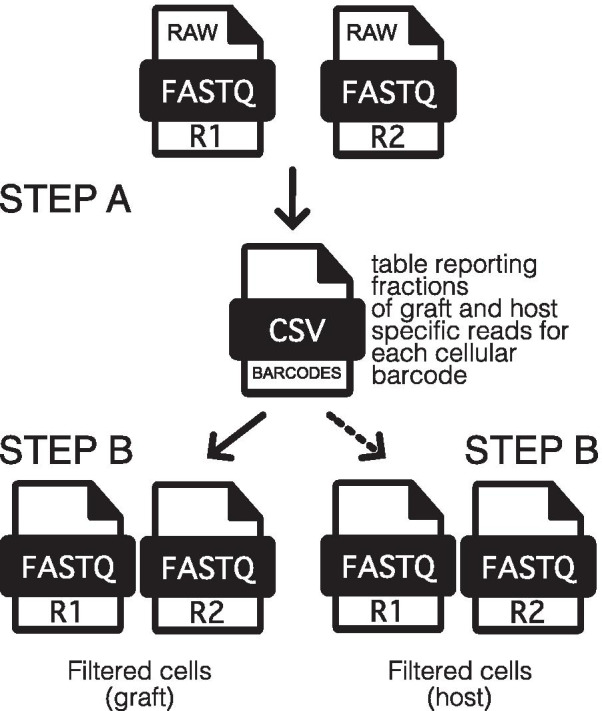
Schematic workflow of XenoCell. The illustration shows the step-wise operations being performed on the input paired FASTQ files from droplet-based single-cell sequencing experiments

The XenoCell procedure consists of two main steps schematically represented in Fig. 1, starting from paired R1 and R2 FASTQ files (input) containing, respectively, barcodes and cDNA sequence from droplet-based single-cell RNA or DNA sequencing experiments.

**Fig. 2:**
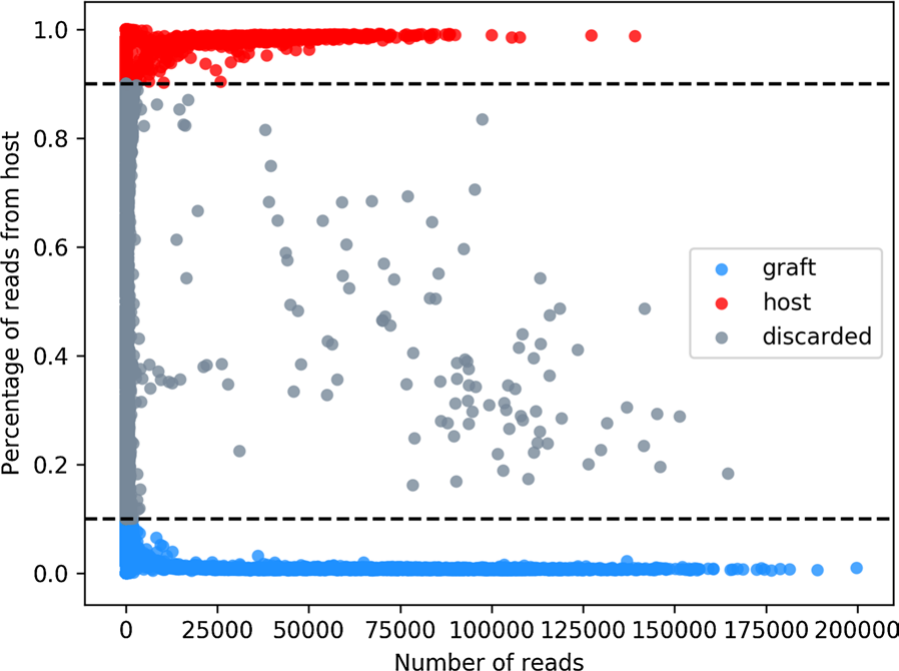
Extracting subsets of cellular barcodes based on specified thresholds. Each dot represents a cellular barcode and its corresponding percentage of reads coming from the host. Depicted dashed lines indicate the thresholds used for XenoCell (0–0.1 for graft-specific cellular barcodes; 0.9–1.0 for host-specific cellular barcodes)

- **Step A** XenoCell classifies each read into one of five classes (graft-specific, host-specific, ambiguous, both, and neither), retrieves the corresponding barcode-containing read for reads unambiguously assigned to either graft or host, and creates a CSV table as an intermediate output. This table contains the fractions of graft- and host-specific reads for each cellular barcode. In this step, XenoCell takes advantage of Xenome [5] to ensure highly accurate classification.
- **Step B** XenoCell extracts graft-specific cellular barcodes based on user-defined upper/lower thresholds for the tolerated fraction of host/graft-specific reads (Fig. 2) (with respect to all the reads associated with a particular cellular barcode).

In a separate (optional) step, users can repeat step B with a different threshold to extract host- specific cellular barcodes, thereby allowing a separate analysis of the host cells.

The final output of XenoCell consists of filtered, paired FASTQ files which are ready to be analysed by any standard bioinformatic pipeline for single-cell analysis, such as Cell Ranger [9] as well as custom workflows, e.g. based on STAR [10], Seurat [11] and scanpy [12].

### Dataset

The mixed-species dataset was generated using the 10× Genomics Chromium Single Cell 3′ Library (v3 chemistry) and then sequenced on Illumina NovaSeq machine. It is available through the 10× Genomics website (https://support.10xgenomics.com/single-cell-gene-expression/datasets/3.0.0/hgmm_5k_v3) and licensed under the Creative Commons Attribution license. The PDX dataset was generated using Drop-seq [13] and it is publicly available through the Gene Expression Omnibus repository (https://www.ncbi.nlm.nih.gov/geo/) under accession number GSE128195 [14].

### scRNA-Seq data analysis

The downstream data analysis of the mixed-species dataset, after XenoCell pre-processing, involved the following steps and relative tools:

- FASTQ files were processed using Cell Ranger v3.0.2, including alignment and cell barcode filtering.
- Resulting count matrices were merged and processed in R v3.5.3 and, among other tools, Seurat v3.0.
- Correlations of transcripts per gene between XenoCell-filtered and unfiltered cells were calculated in R v3.5.3 both for graft and host cells.

The downstream data analysis of the PDX dataset, after XenoCell pre-processing, involved STARsolo v2.7.5c for read alignment, cell barcode filtering, and generation of the transcript count matrix.

### Code

All commands and steps, together with further information regarding the installation process and usage details, are documented and freely available through the XenoCell GitLab repository (https://gitlab.com/XenoCell/XenoCell). We also provide a minimal Snakemake-based [7] XenoCell pipeline that allows to run the previously described steps on multiple samples in an automated and parallelized fashion, ideally performed on High Performance Computing (HPC) clusters due to high memory requirements.

### Runtime assessment

In the 50:50 mixed-species dataset, using 16 CPU cores, read classification (workflow step A) took 2 h 27 m and extraction of graft cells (workflow step B) took 1 h 31 m to finish. In the PDX dataset, using 16 CPU cores, read classification finished within 1 h 40 m, and extraction of graft cells took 1 h 14 m to complete. More details can be found in Additional file 1: Table S1.

## Results and discussion

XenoCell is a Python-based wrapper around Xenome [5] supplied with functions for data processing. An overview of the XenoCell workflow is shown in Fig. 1 (see Implementation section). The input to XenoCell consists of paired reads from droplet-based single-cell sequencing experiments of xenograft samples from any proportion of host/graft species.

Once for, every cellular barcode, the percentage of graft- and host-specific reads is calculated (Step A), the extraction of cellular barcodes representing the cells from the organism of interest is performed (Step B) on the basis of a user-defined threshold of tolerance (Fig. 2).

To assess the performance of XenoCell, we applied it to a publicly available single-cell gene expression dataset released by 10× Genomics (supplementary data), which is composed of a 1:1 mixture of fresh frozen human (HEK293T) and mouse (NIH3T3) cells (for a total of ~ 5000 cells).

We retrieved graft- and host-specific cellular barcodes, containing a maximum of 10% or a minimum of 90% host-specific reads, respectively. Then, we used the XenoCell-filtered FASTQ output files as input for Cell Ranger to align the reads against the hg19 and mm10 genome assemblies, respectively, resulting in 2532 graft and 2626 host cells, reflecting the initial 1:1 mixture of cells.

We compared our results against the well-established Cell Ranger pipeline, which currently is the only one supporting a comparable approach (albeit exclusively applicable to scRNA-seq data generated with the 10× Genomics platform).

We took advantage of a function of Cell Ranger that is able to create a combined reference genome from two species, align reads from a single data set against it, and quantify the fraction of species-specific reads for each cell of the data set, thereby allowing to split the cells by the species they originated from. To compare the assignment of cells to one of two species through XenoCell and Cell Ranger in the aforementioned example data set, we have prepared a combined reference genome made from human (hg19) and mouse (mm10) using this feature of Cell Ranger.

**Fig. 3:**
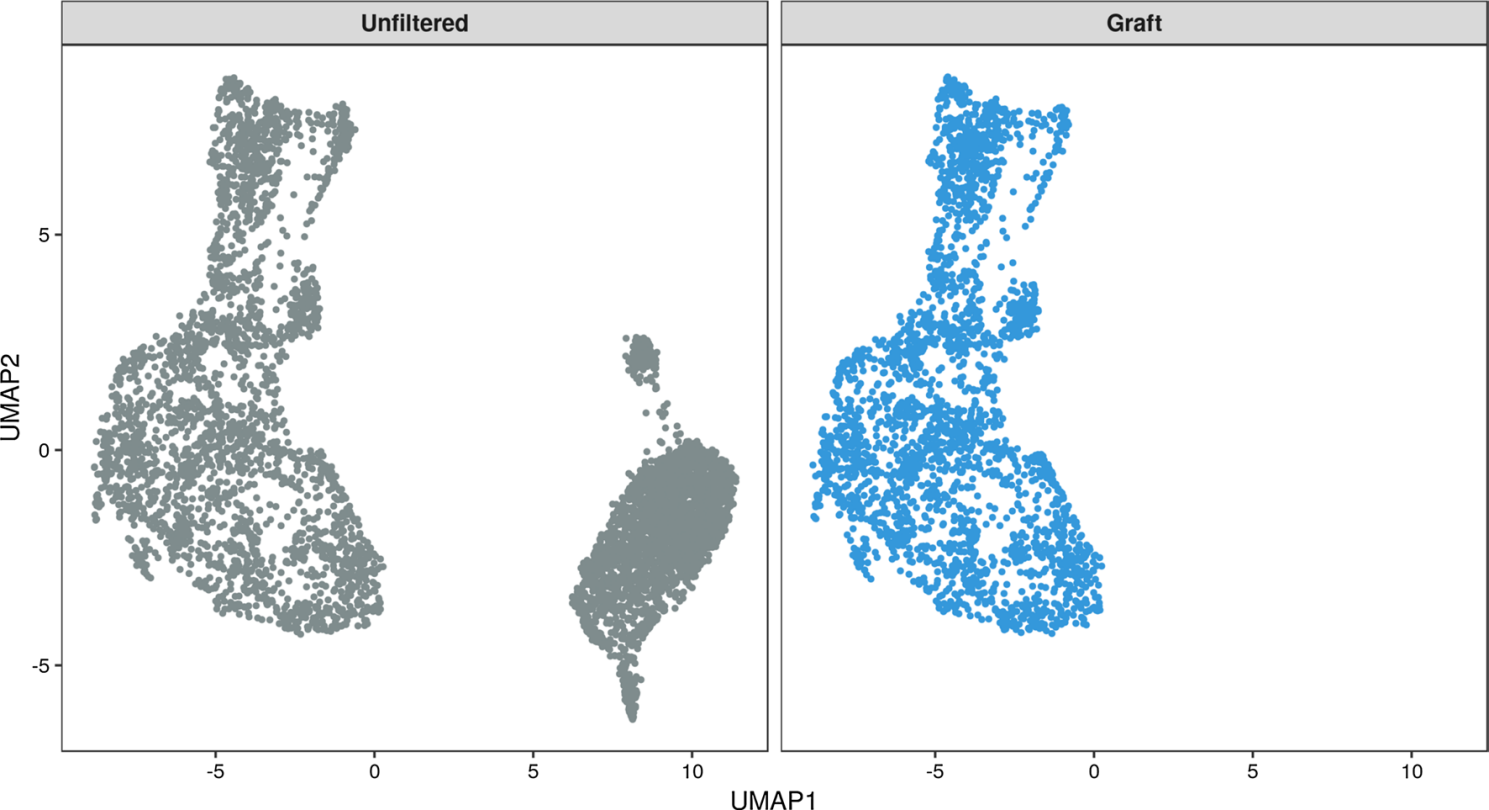
UMAP generated with unfiltered cells and graft-specific cells after alignment to the human genome. As reflected by the full overlap of the graft cells (blue) with the left group of the unfiltered cells (gray), XenoCell successfully extracted only graft cells and, more importantly, without affecting their transcriptomic profiles

**Fig. 4:**
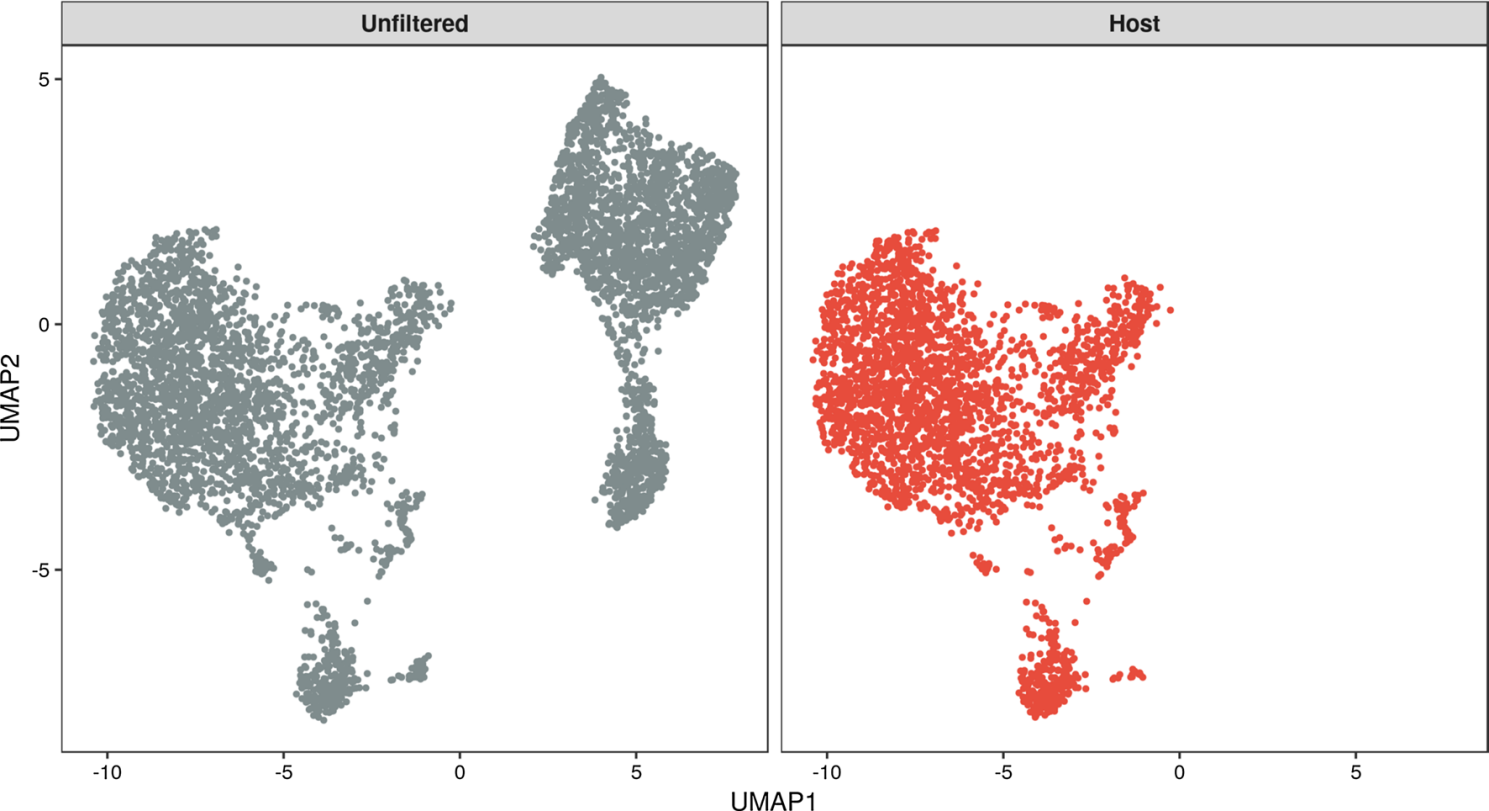
UMAP generated with unfiltered cells and host-specific cells after alignment to the mouse genome. As reflected by the full overlap of the graft cells with the right group of the unfiltered cells (gray), XenoCell successfully extracted only host cells (red) and, more importantly, without affecting their transcriptomic profiles

We performed a total of three analyses: (1) alignment of the unfiltered data set to the combined human-mouse reference genome, (2) alignment of human cells extracted with XenoCell to the human reference genome, (3) alignment of mouse cells extracted with XenoCell to the mouse reference genome. The number of cells identified after XenoCell filtering in analyses 2 and 3 confirm the initial 1:1 proportion of human and mouse cells in the data set. Moreover, ~ 97% and 100% of the cells identified as graft- and host-specific by XenoCell, respectively, were classified concordantly by Cell Ranger (Additional file 2: Fig. S1). To check whether filtering of cellular barcodes with XenoCell affects the transcriptional profiles of the single cells, potentially due to the removal of reads classified as ambiguous by Xenome, we aligned the unfiltered sample and the graft-specific cells retrieved by XenoCell to the human reference genome (hg19) using Cell Ranger, and represented the transcriptional profiles in a UMAP projection generated with Seurat (Fig. 3). Results clearly show that the graft cells retrieved by XenoCell occupy the same transcriptional space as the unfiltered sample, with the other population of cells likely representing mouse cells. Similarly, we aligned the unfiltered sample and host-specific cells retrieved by XenoCell to the mouse reference genome (mm10) using the same procedure as for the human cells (Fig. 4) thus obtaining the same results. As a confirmation, we measured the correlation of transcripts per gene between XenoCell-filtered and unfiltered cells, for both graft (hg19, r = 1) and host (mm10, r = 1) cells (Additional file 3: Fig. S2). Results lead to the same conclusion that XenoCell does not introduce a systematic bias to the transcriptional profiles of the investigated cells due to the removal of reads which cannot be unambiguously assigned to either human (graft) or mouse (host). Moreover, in both cases, we investigated whether the removed cells (visually identifiable in the UMAP projection of the unfiltered sample as the grey cluster that is not present in the graft and host subsets of Figs. 3 and 4, respectively) were correctly assigned to their respective counterpart. As expected, this was the case for 99.7% and 97.9% of cells for graft-specific and for host-specific scenario. The remaining 7 (0.3%) and 47 (2.1%) cells were found to have significant UMI counts for both organisms (range 16–87%), therefore they were correctly discarded by the imposed thresholds (0–10% of host-specific reads for cellular barcodes from the graft; 90–100% of host-specific reads for cellular barcodes from the host; Additional file 4: Fig. S3).

Overall, XenoCell and the multi-species analysis with Cell Ranger produced concordant results. However, the functionality of Cell Ranger to align reads from scRNA-seq experiments to a multi-species reference genome is only available for samples generated by the 10× Genomics scRNA-seq kits, thereby limiting its applicability. Instead, XenoCell offers the flexibility to set a threshold on the permitted fraction of host/graft-specific reads, depending on the biological question the user poses, and is not restricted to any particular technology or library preparation kit.

In this light, we assessed the effectiveness of XenoCell on a publicly available PDX dataset [14] generated using the Drop-seq protocol [13]. When we applied XenoCell to this this dataset, we detected 11 cellular barcodes containing more than 250,000 host-specific reads and less than 5% graft-specific reads (Additional file 5: Fig. S4), which would likely pass common cell whitelisting methods, ultimately ending up contaminating the dataset. To confirm our suspicion, we performed three separate analyses: (1) alignment of human cells extracted with XenoCell to the human reference genome, (2) alignment of mouse cells extracted with XenoCell to the mouse reference genome, (3) alignment of the unfiltered data set to the human reference genome. Using STARsolo with default parameters to generate transcript count matrices, and intersecting the sets of identified cells from the different analyses, we found that 19 of the 581 cells (3.3%) identified in the unfiltered dataset belonged to the host and were successfully removed by XenoCell in the purified graft sample.

These results suggest that upstream biochemical or physical strategies to purify xenograft samples by removing contaminating cells from the host organism might not always be fully successful, and, therefore, bioinformatic pre-processing of the sequenced data should become a routine practice in single-cell experiments from xenografts. Overall, we demonstrated that XenoCell can be applied to data generated by multiple single-cell technologies and we tested its effectiveness on a mixed-species dataset and a real PDX dataset.

## Conclusion

XenoCell is the first stand-alone tool that is able to classify and separate cellular barcodes in droplet-based single-cell sequencing experiments from xenograft samples. It has a broad range of applications both in terms of single-cell multi-omic data types (including scRNA, scDNA, scChIP, scATAC) and of single-cell protocols (including 10× Genomics Chromium, Drop-Seq [13], inDrop [15], Seq-Well [16], CEL-seq2 [17], MARS-seq/MARS-seq2 [18, 19], mcSCRB-seq [20]), from any combination of host and graft species. XenoCell provides paired FASTQ files as outputs, granting substantial flexibility for further analysis. In conclusion, the proposed tool addresses the urgent needs of software support for analyses of single-cell data.

## Availability and requirements

- Project Name: XenoCell.
- Project home page: https://gitlab.com/XenoCell/XenoCell.
- Operating system(s): Platform-independent.
- Programming language: Python.
- Other requirements: None.
- License: MIT License.
- Any restrictions to use by non-academics: None.

## Supplementary Information

**Additional file 1. Table S1**. Computational resources needed  to process public datasets. The table reports details regarding the setting of the analyzed datasets: 50:50 cell line mixed-species and a published Drop-Seq dataset from a real PDX scRNAseq experiment. Computation time for each step is specified.

**Additional file 2. Fig. S1.** Comparison of barcode classification by XenoCell and Cell Ranger on a mixed human-mouse dataset. Both tools extract mostly the same human cells (~ 97% overlap), with only a few cells specific to each tool. Instead, all murine cells extracted by XenoCell were also found by Cell Ranger. The classification of cellular barcodes which were extracted by both XenoCell and Cell Ranger are concordant in all cases.

**Additional file 3. Fig. S2.** Correlation of transcripts per gene in XenoCell-filtered and unfiltered cells. The plots depict the perfect correlation of transcripts per genes counts calculated for both graft (hg19, left panel) and host (mm10, right panel) cells before and after XenoCell processing.

**Additional file 4. Fig. S3.** Characterization of cellular barcodes missed by XenoCell. Highlighted in red are the 54 (47 + 7) cellular barcodes that were missed after XenoCell preprocessing, which instead were expected to be scored according to the UMAP projection of the unfiltered sample. The plot shows that these 54 cellular barcodes were discarded because they contained high transcript counts for both organisms and were filtered out due to the imposed thresholds. Moreover, we observed 105 cellular barcodes that appear to be hybrid (> 10,000 transcripts, between 10-90% of host-specific reads). The multiplet rate specified by 10× Genomics is expected to be 0.8% in 1000 cells, which is in accordance with our results consisting of 5000 cells and having a chance of cross-species droplet of 50% (cells of the two species were mixed in equal fraction).

**Additional file 5. Fig. S4.** Classification of cellular barcodes in the PDX dataset. The sequenced reads of the P3 sample in the public PDX dataset were classified and grouped by the cellular barcodes they are associated with. Then, cellular barcodes are plotted based on the number of graft- and host-specific reads they contain. A cellular barcode was labelled “graft” if at least 90% of its reads are graft-specific, and “host” if at least 90% of reads are host-specific. While most cellular barcodes clearly originate from graft cells, we identified several cellular barcodes with a high number of host-specific reads, therefore likely originating from host cells that contaminated the sample. While the precise number of contaminating host cells depends on the cell identification threshold, there are 11 cellular barcodes with more than 250,000 host-specific reads and less than 5% graft-specific reads, which would likely pass most cell filtering procedures and be included in the analysis. Without prior XenoCell filtering, 19 host cells were included in the final dataset when we processed it with STARsolo (default parameters).

## Data Availability

The mixed-species dataset that was analyzed in the present study is available through the 10× Genomics website (https://support.10xgenomics.com/single-cell-gene-expression/datasets/3.0.0/hgmm_5k_v3) and licensed under the Creative Commons Attribution license. The raw data of PDX dataset is available at the Sequence Read Archive (https://www.ncbi.nlm.nih.gov/sra) with accession SRP188227 (BioProject: PRJNA526715, GEO: GSE128195).

## Abbreviations

NGS: Next generation sequencing
PDX: Patient-derived xenograft
sc: Single-cell
scATAC: Single-cell assay for transposase-accessible chromatin sequencing
scChIP: Single-cell chromatin immunoprecipitation sequencing
scCNV: Single copy number variants sequencing
scDNA: Single-cell DNA-sequencing
scRNA: Single-cell RNA-sequencing
