# Supplementary material for: XenoCell: classification of cellular barcodes in single cell experiments from xenograft samples
Source: BMC Med Genomics. 2021 Jan 29;14:34. doi: 10.1186/s12920-021-00872-8 (PMC7847033; doi:10.1186/s12920-021-00872-8)
Supplement: Supplementary file 1 — Additional file 1. Table S1. Computational resources needed to process public datasets. The table reports details regarding the setting of the analyzed datasets: 50:50 cell line mixed-species and a published Drop-Seq dataset from a real PDX scRNAseq experiment. Computation time for each step is specified. [file 12920_2021_872_MOESM1_ESM.pdf]

Table S1

		Mixed-species dataset	Public PDX dataset
Technology		10x 5’ v3	Drop-seq
Read lengths (R1 + R2)		28 bp + 91 bp	20 bp + 65 bp
Read containing barcodes		R1	R1
Barcode layout		16 bp cellular barcode + 12 bp UMI	12 bp cellular barcode + 8 bp UMI
Number of reads		313 million	268 million
Number of cells		~ 5,000	~ 550
Fraction of host cells		~ 50%	~ 3.5%
Computation time (16 threads)	Classify reads	2h 27min	1h 40min
	Extract graft cells	1h 31min	1h 14min
	Extract host cells	1h 8min	37min

Table S1: **Computational resources needed to process public datasets.** The table reports details regarding the setting of the analyzed datasets: 50:50 cell line mixed-species and a published Drop-Seq dataset from a real PDX scRNAseq experiment. Computation time for each step is specified.
