# Supplementary material for: XenoCell: classification of cellular barcodes in single cell experiments from xenograft samples
Source: BMC Med Genomics. 2021 Jan 29;14:34. doi: 10.1186/s12920-021-00872-8 (PMC7847033; doi:10.1186/s12920-021-00872-8)
Supplement: Supplementary file 2 — Additional file 2. Fig. S1. Comparison of barcode classification by XenoCell and Cell Ranger on a mixed human-mouse dataset. Both tools extract mostly the same human cells (~ 97% overlap), with only a few cells specific to each tool. Instead, all murine cells extracted by XenoCell were also found by Cell Ranger. The classification of cellular barcodes which were extracted by both XenoCell and Cell Ranger are concordant in all cases. [file 12920_2021_872_MOESM2_ESM.pdf]

Fig. S1

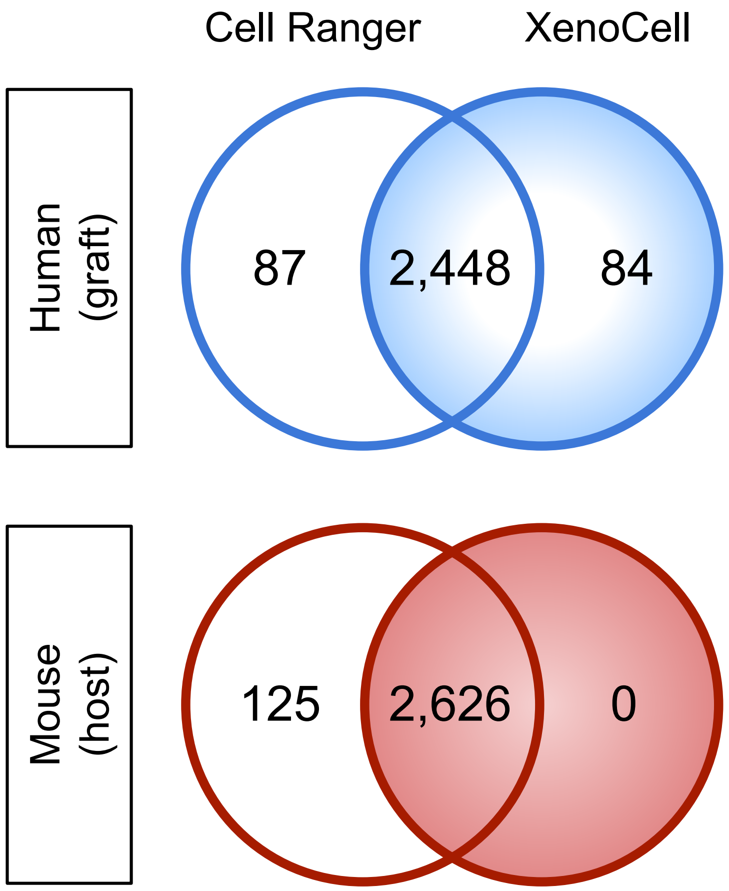

Fig. S1: **Comparison of barcode classification by XenoCell and Cell Ranger on a mixed human-mouse dataset.** Both tools extract mostly the same human cells (~97% overlap), with only a few cells specific to each tool. Instead, all murine cells extracted by XenoCell were also found by Cell Ranger. The classification of cellular barcodes which were extracted by both XenoCell and Cell Ranger are concordant in all cases.
