# Supplementary material for: XenoCell: classification of cellular barcodes in single cell experiments from xenograft samples
Source: BMC Med Genomics. 2021 Jan 29;14:34. doi: 10.1186/s12920-021-00872-8 (PMC7847033; doi:10.1186/s12920-021-00872-8)
Supplement: Supplementary file 3 — Additional file 3. Fig. S2. Correlation of transcripts per gene in XenoCell-filtered and unfiltered cells. The plots depict the perfect correlation of transcripts per genes counts calculated for both graft (hg19, left panel) and host (mm10, right panel) cells before and after XenoCell processing. [file 12920_2021_872_MOESM3_ESM.pdf]

Fig S2

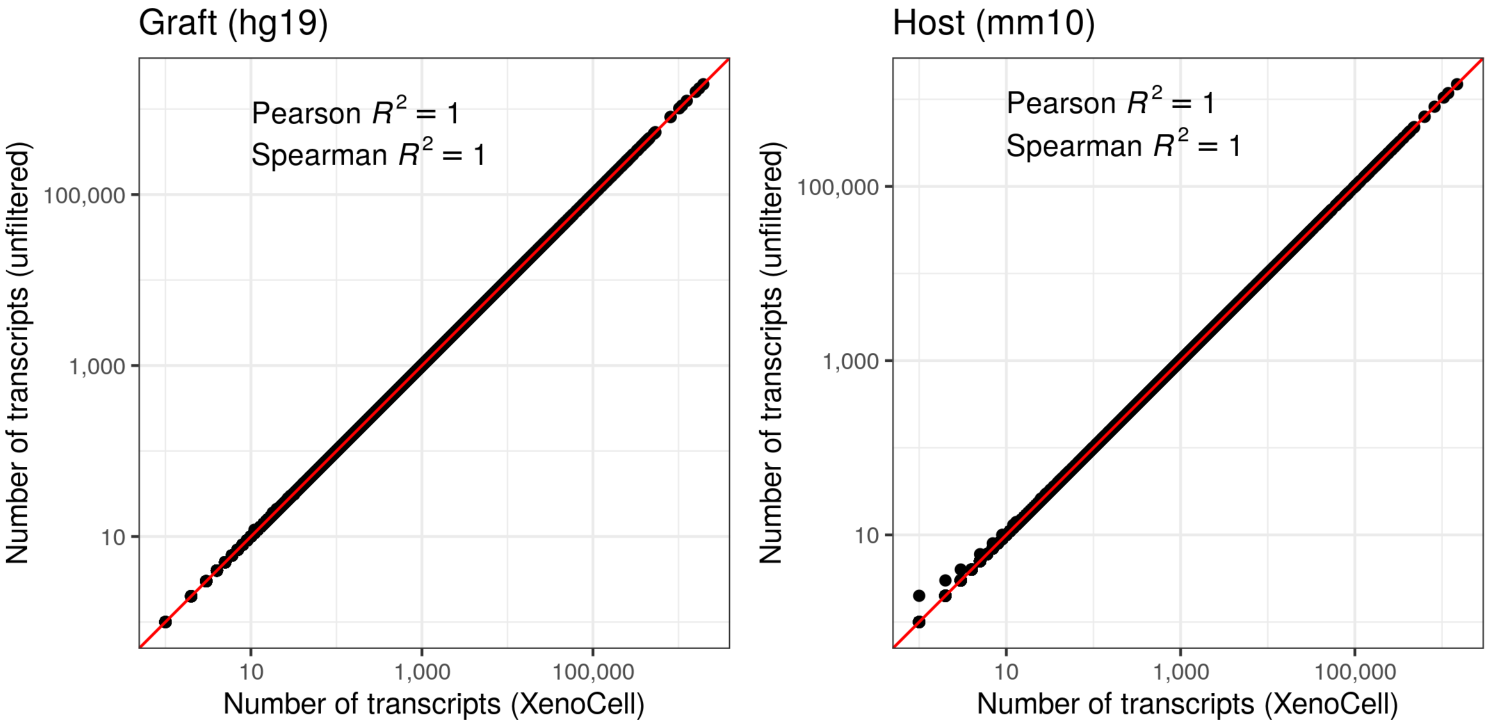

Fig. S2: **Correlation of transcripts per gene in XenoCell-filtered and unfiltered cells.** The plots depict the perfect correlation of transcripts per genes counts calculated for both graft (hg19, left panel) and host (mm10, right panel) cells before and after XenoCell processing.
