# Supplementary material for: XenoCell: classification of cellular barcodes in single cell experiments from xenograft samples
Source: BMC Med Genomics. 2021 Jan 29;14:34. doi: 10.1186/s12920-021-00872-8 (PMC7847033; doi:10.1186/s12920-021-00872-8)
Supplement: Supplementary file 4 — Additional file 4. Fig. S3. Characterization of cellular barcodes missed by XenoCell. Highlighted in red are the 54 (47 + 7) cellular barcodes that were missed after XenoCell preprocessing, which instead were expected to be scored according to the UMAP projection of the unfiltered sample. The plot shows that these 54 cellular barcodes were discarded because they contained high transcript counts for both organisms and were filtered out due to the imposed thresholds. Moreover, we observed 105 cellular barcodes that appear to be hybrid (> 10,000 transcripts, between 10-90% of host-specific reads). The multiplet rate specified by 10× Genomics is expected to be 0.8% in 1000 cells, which is in accordance with our results consisting of 5000 cells and having a chance of cross-species droplet of 50% (cells of the two species were mixed in equal fraction). [file 12920_2021_872_MOESM4_ESM.pdf]

Fig. S3

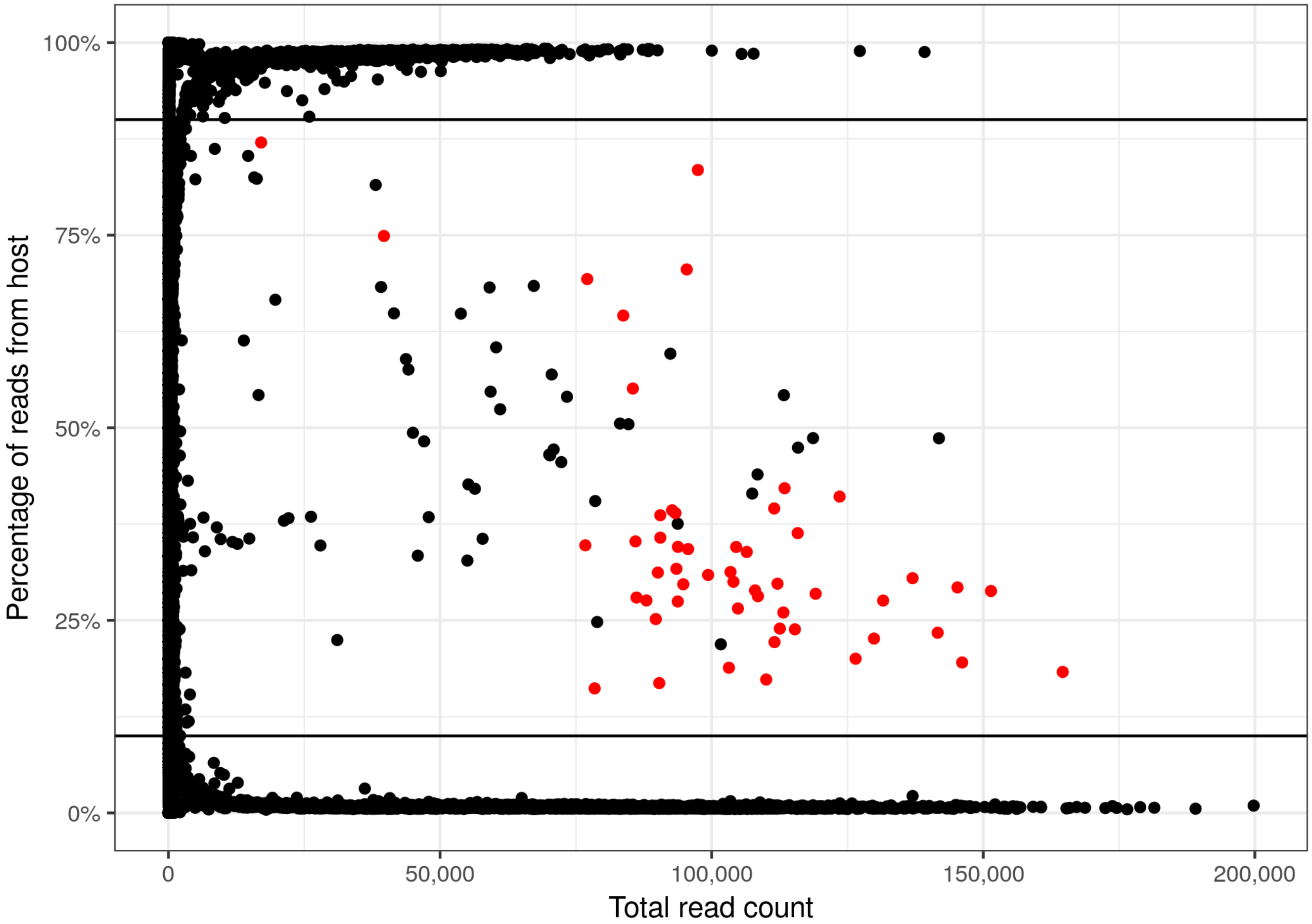

Fig. S3: **Characterization of cellular barcodes missed by XenoCell.** Highlighted in red are the 54 (47 + 7) cellular barcodes that were missed after XenoCell preprocessing, which instead were expected to be scored according to the UMAP projection of the unfiltered sample. The plot shows that these 54 cellular barcodes were discarded because they contained high transcript counts for both organisms and were filtered out due to the imposed thresholds.

Moreover, we observed 105 cellular barcodes that appear to be hybrid (>10,000 transcripts, between 10-90% of host-specific reads). The multiplet rate specified by 10x Genomics is expected to be 0.8% in 1,000 cells, which is in accordance with our results consisting of 5,000 cells and having a chance of cross-species droplet of 50% (cells of the two species were mixed in equal fraction).
