# Supplementary material for: XenoCell: classification of cellular barcodes in single cell experiments from xenograft samples
Source: BMC Med Genomics. 2021 Jan 29;14:34. doi: 10.1186/s12920-021-00872-8 (PMC7847033; doi:10.1186/s12920-021-00872-8)
Supplement: Supplementary file 5 — Additional file 5. Fig. S4. Classification of cellular barcodes in the PDX dataset. The sequenced reads of the P3 sample in the public PDX dataset were classified and grouped by the cellular barcodes they are associated with. Then, cellular barcodes are plotted based on the number of graft- and host-specific reads they contain. A cellular barcode was labelled “graft” if at least 90% of its reads are graft-specific, and “host” if at least 90% of reads are host-specific. While most cellular barcodes clearly originate from graft cells, we identified several cellular barcodes with a high number of host-specific reads, therefore likely originating from host cells that contaminated the sample. While the precise number of contaminating host cells depends on the cell identification threshold, there are 11 cellular barcodes with more than 250,000 host-specific reads and less than 5% graft-specific reads, which would likely pass most cell filtering procedures and be included in the analysis. Without prior XenoCell filtering, 19 host cells were included in the final dataset when we processed it with STARsolo (default parameters). [file 12920_2021_872_MOESM5_ESM.pdf]

Fig. S4

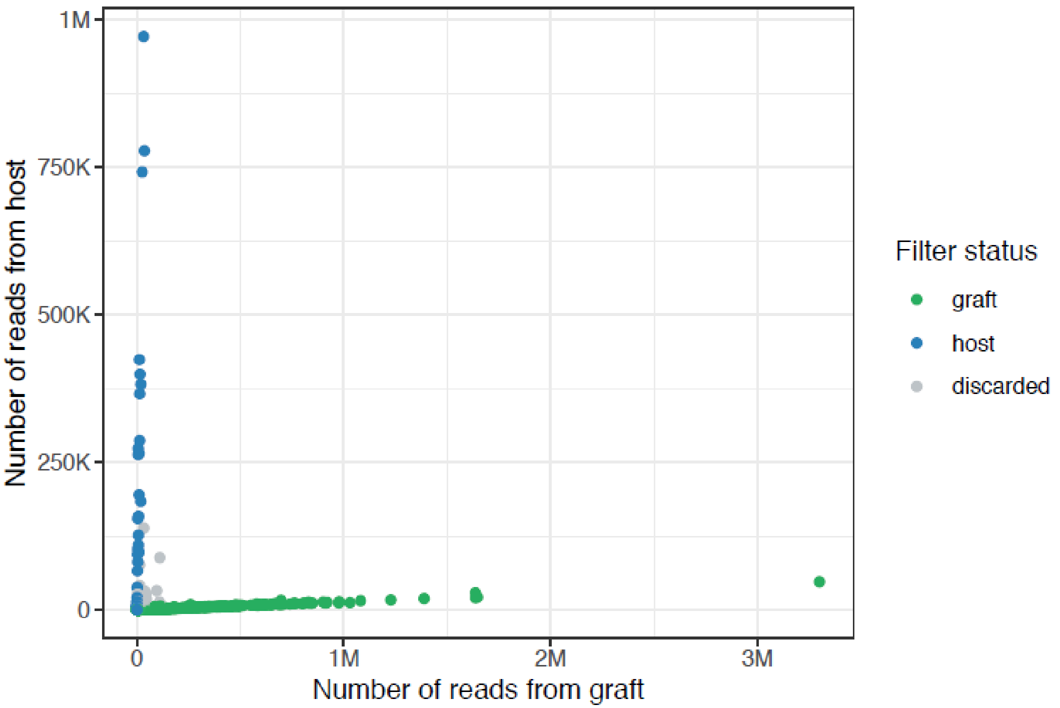

Fig. S4: **Classification of cellular barcodes in the PDX dataset.** The sequenced reads of the P3 sample in the public PDX dataset were classified and grouped by the cellular barcodes they are associated with. Then, cellular barcodes are plotted based on the number of graft- and host-specific reads they contain. A cellular barcode was labelled “graft” if at least 90% of its reads are graft-specific, and “host” if at least 90% of reads are host-specific. While most cellular barcodes clearly originate from graft cells, we identified several cellular barcodes with a high number of host-specific reads, therefore likely originating from host cells that contaminated the sample. While the precise number of contaminating host cells depends on the cell identification threshold, there are 11 cellular barcodes with more than 250,000 host-specific reads and less than 5% graft-specific reads, which would likely pass most cell filtering procedures and be included in the analysis. Without prior XenoCell filtering, 19 host cells were included in the final dataset when we processed it with STARsolo (default parameters).
